# Bleeding anorectal varices treated by a direct puncture approach through the greater sciatic foramen: The utility of a steerable microcatheter for reverse catheterization^a^

**DOI:** 10.1016/j.radcr.2022.01.051

**Published:** 2022-02-03

**Authors:** Shohei Chatani, Kokichi Seki, Akinaga Sonoda, Yoko Murakami, Yuki Tomozawa, Takehide Fujimoto, Akira Andoh, Yoshiyuki Watanabe

**Affiliations:** a Department of Radiology, Shiga University of Medical Science, Seta, Tsukinowa-cho, Otsu, Shiga, 520-2192, Japan; b Department of Gastroenterology, Shiga University of Medical Science, Seta, Tsukinowa-cho, Otsu, Shiga, 520-2192, Japan

**Keywords:** Anorectal varices, Ectopic varices, Interventional radiology, Embolization, Steerable, Microcatheter

## Abstract

Bleeding is less common from anorectal varices than from esophageal varices, but it is potentially life-threatening. Here, we present a case of a woman in her 70s with critical hemorrhage from anorectal varices. The endoscopic approach could not be performed due to the huge variceal formation and the transhepatic approach was also unsuitable due to the presence of portal vein thrombosis and ascites. A direct puncture to the right superior rectal vein was performed through the greater sciatic foramen under computed tomography fluoroscopic guidance. Using a steerable microcatheter, superior rectal veins were bilaterally embolized with a mixture of n-butyl cyanoacrylate and ethiodized oil, and microcoils. Endoscopy and contrast-enhanced computed tomography performed after the procedure confirmed a marked shrinkage of anorectal varices. When endoscopic or any other approaches are difficult, this technique can be a useful alternative therapeutic option.

## Introduction

Anorectal varices represent a developed portosystemic collateral pathway where portal venous blood flows from the superior rectal veins of the inferior mesenteric system to the middle and inferior rectal veins of the iliac system [1,2]. Anorectal varices most commonly result from portal hypertension, and they occur with an incidence of 38%-92% in patients with liver cirrhosis [3,4]. Bleeding from anorectal varices is less common than from esophageal varices, but it can be massive and may become life-threatening. In contrast to the management of esophageal varices, there are no established guidelines to define the appropriate treatment for anorectal varices.

Here, we report a case of life-threatening hemorrhaging from intractable anorectal varices, which was successfully treated by embolization of bilateral superior rectal veins delivered through a steerable microcatheter. However, the catheter, by necessity, had to be guided through a remarkably unusual path—with direct puncture access to the right superior rectal vein approached through the greater sciatic foramen. This technically demanding but feasible approach can be a lifesaving alternative therapeutic option to treat hemorrhaging anorectal varices in cases where endoscopic or any other approaches are difficult or contraindicated.

## Case report

### History

**Fig. 1:**
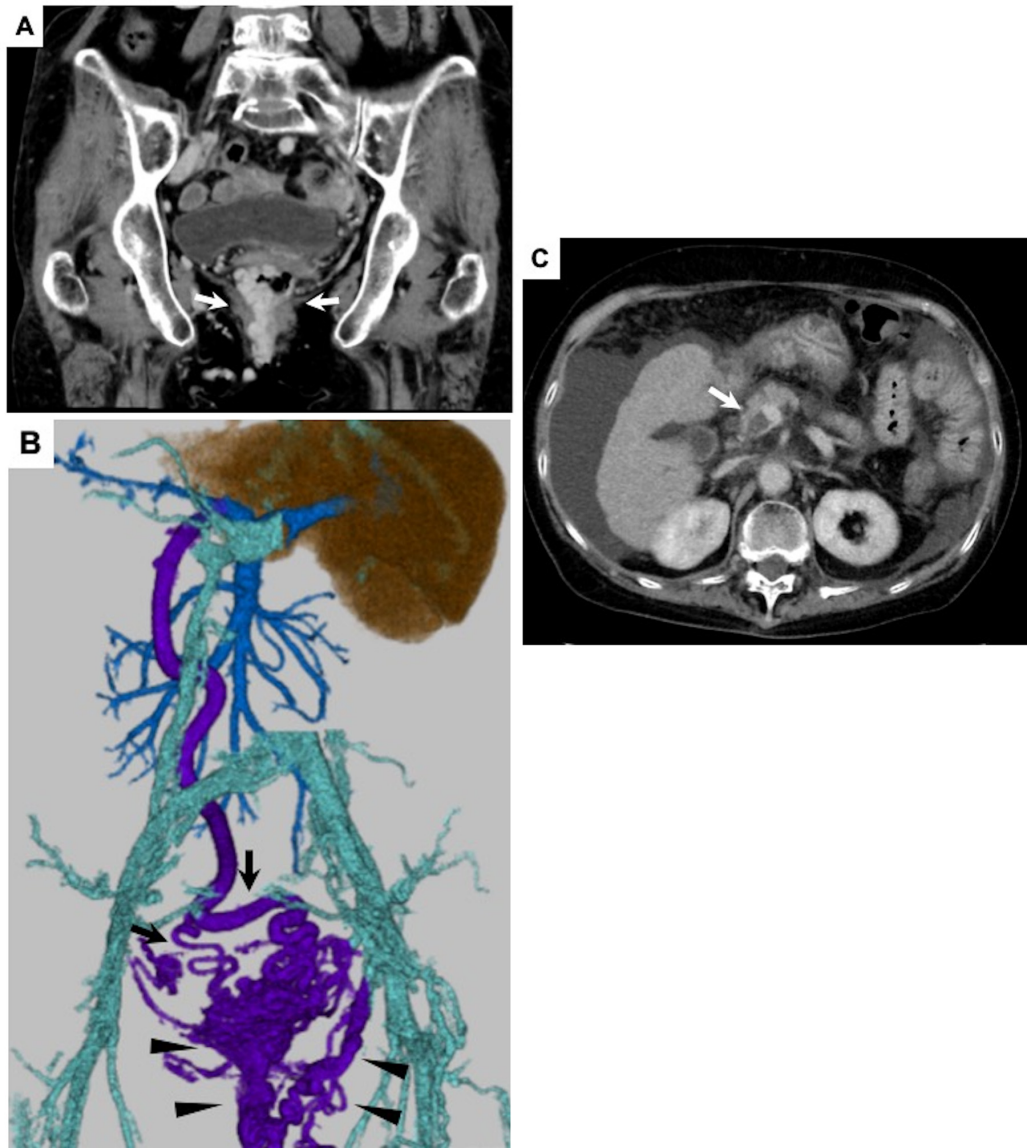
(A) Contrast-enhanced computed tomography (CECT) in coronal view revealed huge anorectal variceal formation (arrow). (B) Volume rendering of CECT (rear view) showed portosystemic collateral pathway where portal venous blood flowed from the bilateral superior rectal veins (arrow) to the anorectal varices with multiple draining veins (arrowhead). (C) CECT in axial view depicted severe liver cirrhosis with portal vein thrombosis (arrow) and ascites.

The patient was a woman in her 70s with Child-Pugh class B primary biliary cholangitis. She was admitted to our hospital for treatment of intractable anorectal varices. Her gastroesophageal varices were repeatedly treated at another hospital, 5 and 2 years ago, respectively, by performing balloon-occluded retrograde transvenous obliteration (BRTO), endoscopic injection sclerotherapy (EIS), and endoscopic band ligation (EBL). Hemorrhage from anorectal varices occurred frequently and contrast-enhanced computed tomography (CECT) revealed large variceal formation in the anorectum (Fig. 1A, B). Portal vein thrombosis was also detected (Fig. 1C), and because it was considered to be a factor exacerbating her anorectal varices, anticoagulant therapy was introduced for treatment. However, a massive hemorrhage from the anorectal varices occurred again and the patient developed hemorrhagic shock (blood pressure, 62/47 mm Hg; heart rate, 110 beats per minute). The endoscopic approach was considered to be difficult due to the overdeveloped variceal formation and, instead, the interventional radiology (IR) approach was planned. Laboratory data obtained just before the procedure indicated severe anemia (hemoglobin, 6.9 g/dL), moderate thrombocytopenia (platelets, 113,000/μL), prolonged coagulation time (prothrombin-international normalized ratio, 1.31) and normal kidney function (creatinine, 0.97 mg/dL).

### Direct puncture access to the right superior rectal vein approached through the greater sciatic foramen

**Fig. 2:**
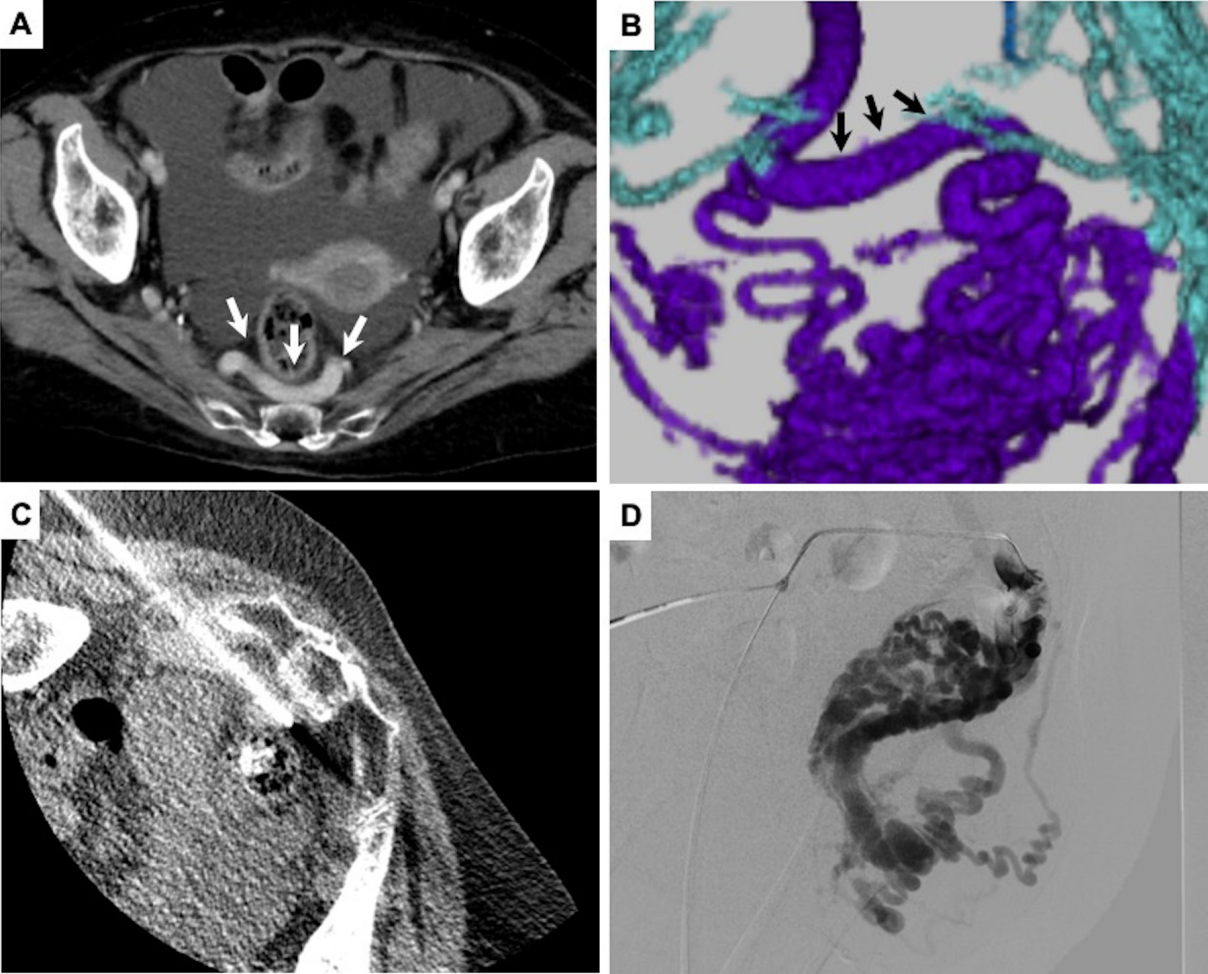
Contrast-enhanced computed tomography (CECT) revealed the dilatation of the right superior rectal vein (arrow) in (A) (axial view) and (B) (rear view of volume rendering). (C, A) direct puncture to the right superior rectal vein under CT fluoroscopic guidance. (D) Digital subtraction angiography from the right superior rectal vein showed large anorectal varices***.***

BRTO appeared unsuitable because multiple draining veins were involved. Transjugular intrahepatic portosystemic shunt (TIPS) and percutaneous transhepatic obliteration (PTO) were not possible due to the presence of portal vein thrombosis and ascites. In CECT, dilatation of the right superior rectal vein which mainly supplied the varices was observed (Fig. 2A, B). For these reasons, we decided to perform obliteration of the varices by direct puncture access to this vein approached through the left greater sciatic foramen.

The procedure was performed using a hybrid interventional radiography/computed tomography (IVR-CT) system (Infinix Celeve-i+Aquilion ONE, Canon Medical Systems, Tochigi, Japan). In the prone oblique position, local anesthesia (1% lidocaine) was administered, and the right superior rectal vein was punctured with an 18-gauge needle under CT fluoroscopic guidance (Fig. 2C). Subsequently, a 0.035-inch guidewire (Radiofocus, Terumo, Tokyo, Japan) was inserted toward the feeding route of the varices and a 5F sheath introducer (Super Sheath, Medikit, Tokyo, Japan) was placed at the right superior rectal vein under fluoroscopic guidance (Fig. 2D).

### Embolization of bilateral superior rectal veins using a steerable catheter

**Fig. 3:**
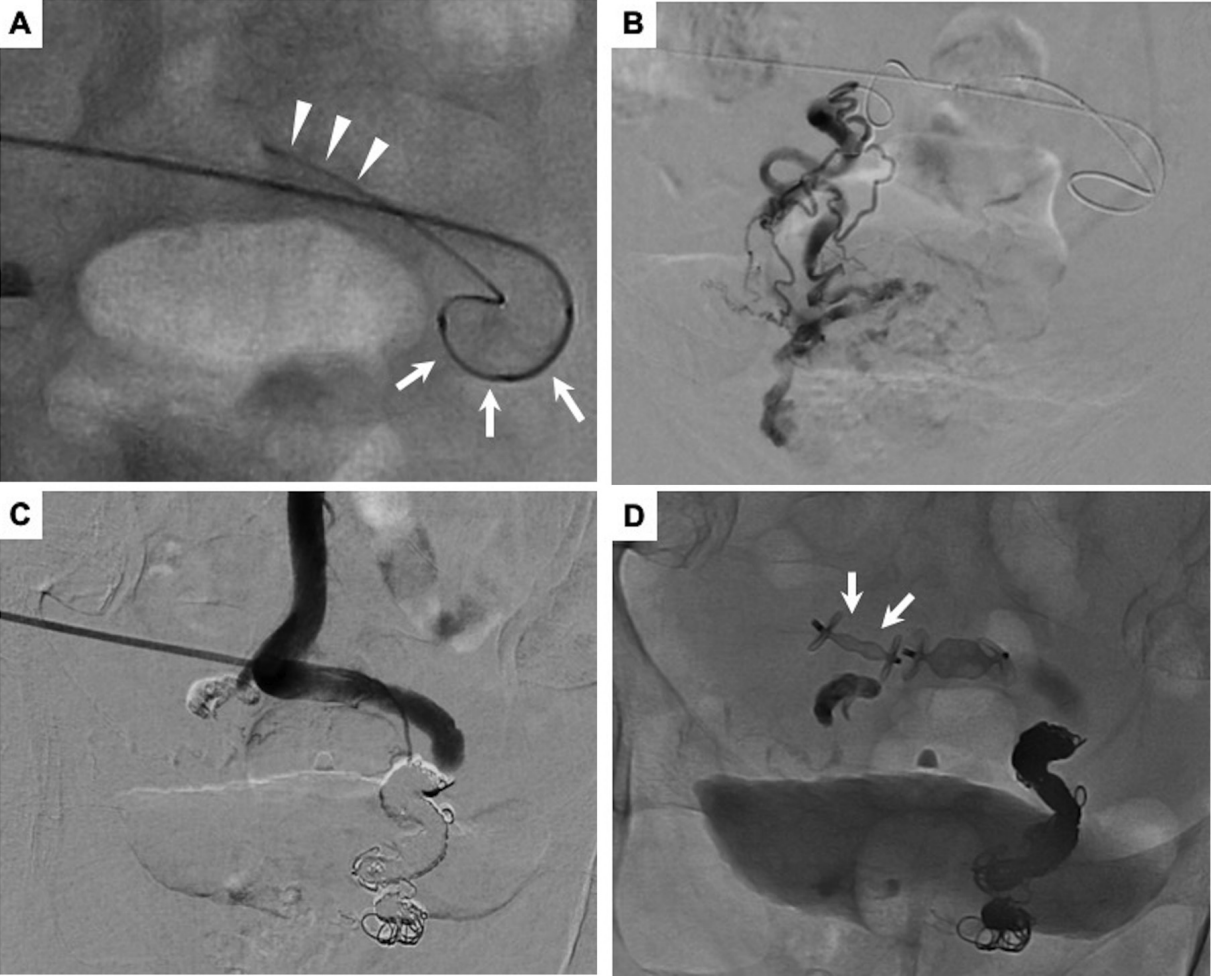
(A) Reverse catheterization using a steerable microcatheter (arrow) and a coaxially inserted microcatheter (arrowhead). (B) Digital subtraction angiography (DSA) from the left superior rectal vein. (C) DSA after embolization of bilateral superior rectal veins showed the disappearance of anorectal varices and normalized hepatopetal flow of inferior mesenteric vein. (D) The puncture tract was embolized using plugs (arrow).

Although the sheath introducer was placed at the right superior rectal vein, we needed to access the left superior rectal vein as well, as it also fed the varices. Therefore, a steerable microcatheter (2.9F distal, 2.9F proximal external diameter; Swift NINJA, Sumitomo Bakelite, Tokyo, Japan) was inverted from the right superior rectal vein, and then a smaller microcatheter (1.9F distal, 1.9F proximal external diameter; Carnelian MARVEL, Tokai Medical Products, Aichi, Japan) was advanced coaxially into the left superior rectal vein (Fig. 3A). Digital subtraction angiography (DSA) from the left superior rectal vein depicted a relatively slow blood flow supplying the anorectal varices (Fig. 3B). Then obliteration was conducted using a 1:4 (v/v) mixture of n-butyl cyanoacrylate (NBCA) (Histoacryl, B. Braun Melsungen AG, Melsungen, Germany) and ethiodized oil (Lipiodol, Guerbet, Villepinte, France).

Next, the inserted smaller microcatheter was advanced to the right superior rectal vein after moving the tip of a steerable microcatheter. Although balloon-occluded antegrade venography was performed, blood flow into the systemic circulation through varices did not disappear due to the complicated and high-flow shunt formation. Thus, to avoid nontarget embolization, obliteration using liquid materials was not conducted. Instead, we proceeded to occlude several feeding veins with detachable coils (Target XL, Stryker, Fremont, CA; Ruby coil 400 and POD packing coil, Penumbra Inc., Alameda, CA). DSA after embolization showed the disappearance of anorectal varices and normalized hepatopetal flow in the inferior mesenteric vein (Fig. 3C). The puncture tract was embolized with plugs (Amplatzer vascular plug Ⅱ, Abbott Vascular, Santa Clara, CA) (Fig. 3D).

### Clinical course after embolization

**Fig. 4:**
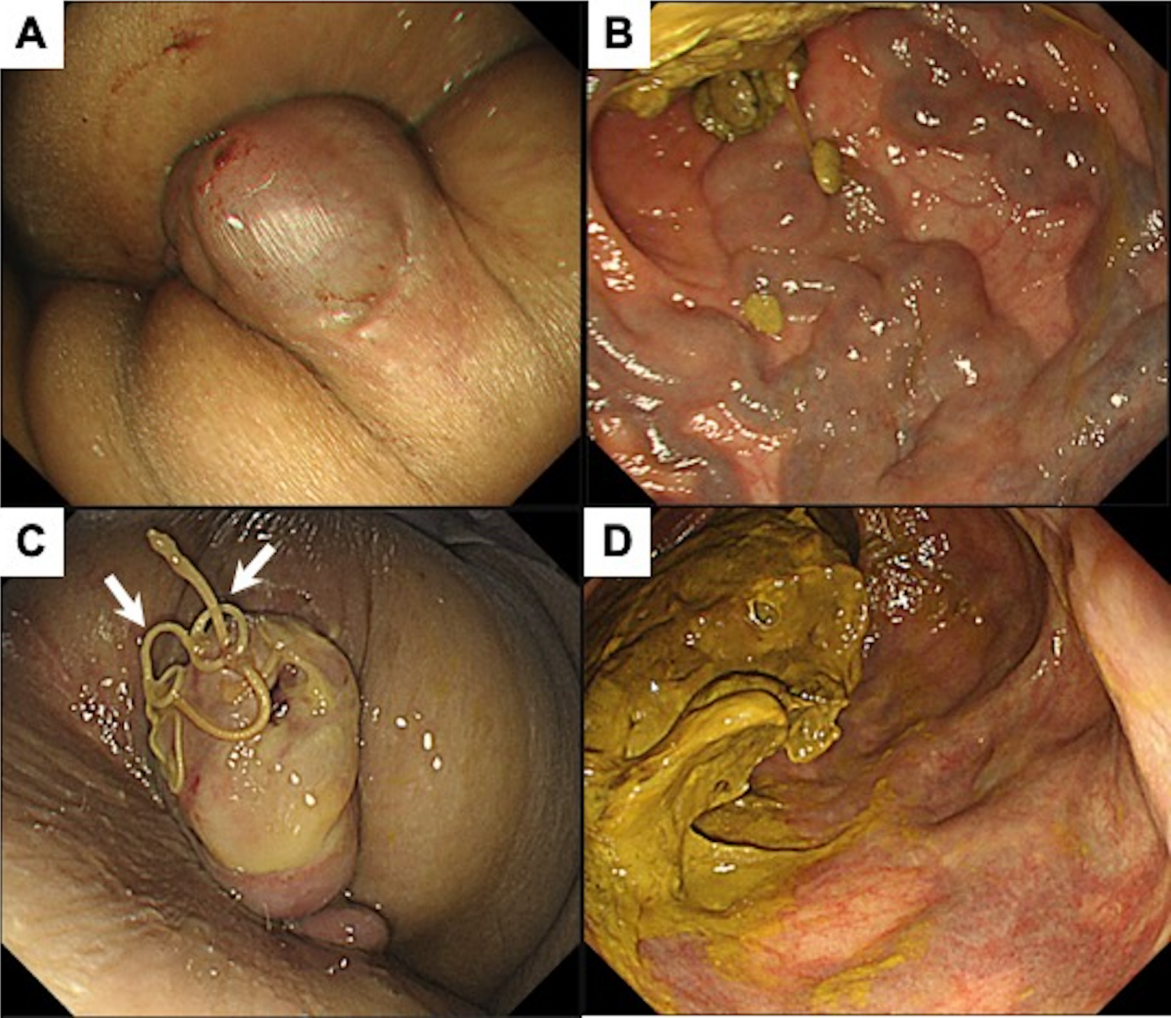
Endoscopic images taken (A, B) before and (C, D) after treatment. The ligature thread used for surgical ligation is indicated (arrow).

Endoscopy, which was performed 2 days after the procedure, confirmed a marked shrinkage of anorectal varices (Fig. 4; compare A and B “before” vs C and D “after”). Furthermore, CECT revealed occlusion of anorectal varices. However, CT also revealed a thrombus of the inferior mesenteric vein, and exacerbation of ascites was observed. Bleeding from esophageal varices occurred 2 weeks after the procedure, and it was treated by EBL. The patient is being carefully followed up for ascites control and thrombolytic therapy for portal vein thrombosis.

## Discussion

Bleeding from rectal varices has a considerably high incidence (37%) [5]. It can be managed by using a variety of methods, including endoscopic therapy (EIS and EBL), IR approach, and surgical treatment, but no standard treatment has been established yet [2,6]. In our case, large, dilated varices rendered both endoscopic options unsuitable. EIS was considered dangerous because, given the high blood flow, injecting sclerosant there could result in pulmonary embolism. EBL was considered difficult to perform because of the size of varices.

IR approaches include TIPS and PTO. However, BRTO is difficult to perform in cases with varices, like ours, where multiple drainage veins are involved. TIPS and PTO cannot be conducted in cases, like ours, where ascites and portal vein thrombosis are present. Direct puncture access to the superior rectal vein approached through the greater sciatic foramen was pioneered by Kariya et al. [7,8], who first used this pelvic approach for successful endovascular treatment for rectal varices and reported its efficacy and feasibility. Although this approach has risks of sciatic nerve injury and vascular damage, it is considered to be safe [9].

Our main original contribution is utilizing the pelvic approach for access with a steerable microcatheter. The steerable microcatheter was recently developed and made commercially available [10,11]. With a dial on the handle, the user controls the bending of the end section that turns the catheter tip in the desired heading direction, which aids access to and catheterization of vessel branches selectively. A small microcatheter can be coaxially inserted into the high-flow type steerable microcatheter, and its utility in the treatment of portal system as well as arterial system has been reported [12,13]. The novel use case here is that by inverting the steerable microcatheter, we were able to advance the inserted smaller microcatheter to the opposite direction in which the sheath introducer was inserted. Thus, we have demonstrated the feasibility of using a steerable microcatheter to make advancing a coaxially inserted microcatheter in the reverse direction possible on demand.

NBCA mixture was used as an embolic material for tract embolization in the previous report [7,8]. However, we used vascular plug for puncture tract embolization because embolization using liquid embolic material when hepatopetal flow in the inferior mesenteric vein is restored to normal levels might result in uncontrolled off-target embolization. Plugs are used for tract embolization of various organs [14,15,16], and have the advantage that they can be stably placed even in peristaltic organs such as the rectum.

In conclusion, we have reported a case of anorectal variceal hemorrhage, which was treated by obliteration via a direct puncture approach through the greater sciatic foramen. Using a steerable microcatheter enables accessing targets in the opposite direction in which the sheath introducer was inserted, and contributes crucially to the capability of complete embolization of varices. When endoscopic or any other approaches are difficult, this technique can be a useful and lifesaving alternative therapeutic option.

## Patient consent

We certify that we have obtained all appropriate patient consent forms.

In the form the patient has given her consent for her images and other clinical information to be reported in the journal. The patient understands that her name and initial will not be published and due efforts will be made to conseal her identity.
